# Multi-targeted kinase inhibition alleviates mTOR inhibitor resistance in triple-negative breast cancer

**DOI:** 10.1007/s10549-019-05380-z

**Published:** 2019-08-06

**Authors:** Jichao He, Ronan P. McLaughlin, Vera van der Noord, John A. Foekens, John W. M. Martens, Gerard van Westen, Yinghui Zhang, Bob van de Water

**Affiliations:** 1 Division of Drug Discovery and Safety, Leiden Academic Centre for Drug Research, Leiden University, 2300 RA Leiden, The Netherlands; 2 Department of Medical Oncology and Cancer Genomic Netherlands, Erasmus MC Cancer Institute, Erasmus Medical Centre, 3000 CA Rotterdam, The Netherlands

**Keywords:** Multi-kinase inhibitor, mTOR-targeted therapy, Drug resistance, Triple-negative breast cancer (TNBC), Polypharmacology

## Abstract

**Purpose:**

Owing to its genetic heterogeneity and acquired resistance, triple-negative breast cancer (TNBC) is not responsive to single-targeted therapy, causing disproportional cancer-related death worldwide. Combined targeted therapy strategies to block interactive oncogenic signaling networks are being explored for effective treatment of the refractory TNBC subtype.

**Methods:**

A broad kinase inhibitor screen was applied to profile the proliferative responses of TNBC cells, revealing resistance of TNBC cells to inhibition of the mammalian target of rapamycin (mTOR). A systematic drug combination screen was subsequently performed to identify that AEE788, an inhibitor targeting multiple receptor tyrosine kinases (RTKs) EGFR/HER2 and VEGFR, synergizes with selective mTOR inhibitor rapamycin as well as its analogs (rapalogs) temsirolimus and everolimus to inhibit TNBC cell proliferation.

**Results:**

The combination treatment with AEE788 and rapalog effectively inhibits phosphorylation of mTOR and 4EBP1, relieves mTOR inhibition-mediated upregulation of cyclin D1, and maintains suppression of AKT and ERK signaling, thereby sensitizing TNBC cells to the rapalogs. siRNA validation of cheminformatics-based predicted AEE788 targets has further revealed the mTOR interactive RPS6K members (RPS6KA3, RPS6KA6, RPS6KB1, and RPS6KL1) as synthetic lethal targets for rapalog combination treatment.

**Conclusions:**

mTOR signaling is highly activated in TNBC tumors. As single rapalog treatment is insufficient to block mTOR signaling in rapalog-resistant TNBC cells, our results thus provide a potential multi-kinase inhibitor combinatorial strategy to overcome mTOR-targeted therapy resistance in TNBC cells.

**Electronic supplementary material:**

The online version of this article (10.1007/s10549-019-05380-z) contains supplementary material, which is available to authorized users.

## Background

Triple-negative breast cancer (TNBC) constitutes a small subtype (10–20%) of breast cancer, but causes the majority of breast cancer-related deaths [1, 2]. As defined by the absence of ER and PR expression and HER2 overexpression, TNBC is not curable by hormone receptor or HER2-targeted therapies [3]. Furthermore, TNBC is highly heterogeneous. Gene expression profiling has further classified TNBC into six unique molecular subtypes, namely basal-like (BL1 and BL2), mesenchymal (M), mesenchymal stem-like (MSL), immunomodulatory (IM), and luminal androgen receptor-like (LAR) subtype [4]. The TNBC molecular signatures have been explored for targeted therapies in clinical trials, including those targeting receptor tyrosine kinases (RTKs, e.g., EGFR, VEGFR, c-Met), PI3K/AKT, Ras/MAPK, JAK/STAT, cell cycle regulators [5, 6]. Yet, TNBC has not benefited from above mono-targeted therapies so far, due to intrinsic or acquired resistance [6].

The mammalian target of rapamycin (mTOR), a conserved serine/threonine protein kinase, is a central regulator of cell growth and proliferation, by sensing and integrating multiple signals from growth factors and nutrient signals [7, 8]. mTOR hyperactivity is frequently observed in TNBC compared to other breast cancer subtypes and is often correlated with poor prognosis, underpinning the potential of mTOR-targeted therapy for TNBC treatment [9–11]. Although mTOR-targeted interventions, such as rapamycin and its analogs (rapalogs) temsirolimus and everolimus, delay progression and extend survival, patients with TNBC eventually develop resistance to mTOR inhibitors with undesired outcome [9, 12]. Evidence has shown that rapalog treatment could release mTOR negative feedback on upstream kinases and activate compensatory pathways, for instance, PI3K/AKT and MAPK/ERK signaling pathways, thereby bypassing mTOR inhibition [13–15]. This observation underscores the need for alternative combinatorial therapeutic approaches for TNBC treatment.

Since oncogenic pathways incorporate multiple signaling components and axes to promote tumor malignancy, monotherapy may not be sufficient for long-term control of TNBC [9, 13, 16]. Hence, simultaneously targeting different signaling molecules represents a promising strategy to impede tumor growth and progression [8, 17]. Several reports have documented that co-targeting growth factor receptors and mTOR exerts cooperative anti-cancer effects in various cancer types, including TNBC [18–22]. However, these studies focus on a particular combination in the questioned cancer type. Little is known about the interactive kinases involved in rapalog resistance and the mechanisms of the combinatorial effect remain unclear. Here, we systematically screened a broad collection of kinase inhibitors across a large panel of TNBC lines treated with rapamycin. Our data demonstrated that multiple targeted kinase inhibition, for instance, by inhibitor AEE788, sensitizes TNBC cells to various mTOR inhibitors, rapamycin, temsirolimus, and everolimus. Integrated cheminformatics study and siRNA validation revealed additional putative targets of AEE788, which interact closely with mTOR signaling. Most importantly, our study provided an efficacious approach for exploring cancer combination treatment. Moreover, the combinatorial therapy is more effective than single drug application and thus demonstrates a therapeutic advantage over either agents as a monotherapy in TNBC treatment.

## Methods

### Kinase inhibitor library combination screen

One day post seeding into 96-well plates, cells were treated with individual kinase inhibitors alone or combined with rapamycin at 1 µM. After 4-day treatment, proliferation was evaluated by sulforhodamine B (SRB) colorimetric assay [23]. Detailed information on materials and methods can be found in Supplementary file ESM_3.

## Results

### TNBC cell lines are differentially responsive to mTOR inhibitor rapalogs

**Fig. 1:**
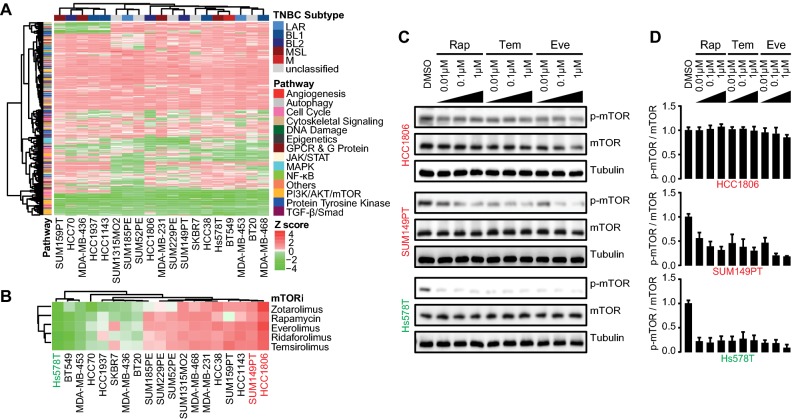
Resistance profiling of TNBC cell lines to mTOR inhibitor rapalogs. **a** Heatmap presenting the responses of 19 TNBC cell lines to 378 kinase inhibitors. Data were shown based on the effect of individual KI on proliferation (relative *Z* scores), subtype-annotated cell lines (clustered horizontally), and pathway-annotated inhibitors (clustered vertically). Strong inhibitory effect on proliferation was indicated in green and weak in red. **b** Response clustering of TNBC cell lines to mTOR inhibitors (mTORi). **c** Concentration range effects of rapalogs rapamycin (Rap), temsirolimus (Tem), and everolimus (Eve) on mTOR phosphorylation, in rapalog-resistant HCC1806 and, SUM149PT TNBC cells, compared to rapalog-sensitive Hs578T cells. Cells were treated with rapalogs in concentration range (µM) for 4 h. **d** Quantitative comparison of phosphorylated mTOR level to total mTOR level in rapalog-treated resistant and sensitive TNBC cells

To gain insights into TNBC dependency on mTOR signaling integration for proliferation and cell survival, a KI library (Selleckchem^®^) containing 378 small molecular inhibitors targeting various kinase signaling pathways was screened across 19 TNBC cell lines (Suppl. Table S1), which are representative for the six transcriptome-based subtypes of TNBC [4]. All TNBC cell lines were exposed to individual inhibitors at 1 µM for 4 days, followed by measurement of cell proliferation. The effect of each inhibitor on proliferation was assessed by *Z* scores normalized to overall proliferative response. TNBC cell lines were largely resistant to the majority of the kinase inhibitors, without any clear correlation to the TNBC molecular subtypes (Fig. 1a). The proliferative response towards mTOR inhibitors was variable among TNBC cell lines. We distinguished 11 TNBC cell lines insensitive to different mTOR inhibitors (Fig. 1b), including rapamycin (Rap) and its analogs (i.e., rapalogs), zotarolimus, everolimus, ridaforolimus, and temsirolimus. HCC1806 and SUM149PT were most resistant to rapologs, while Hs578T was most sensitive.

Rapalogs are highly selective allosteric inhibitors of mTOR, by binding to FKBP12/rapamycin-binding domain to block mTOR Ser2448 phosphorylation and function [24, 25]. mTOR Ser2448 is a predominant phosphorylation residue for mTOR kinase activity in response to mitogen-derived stimuli [25]. Therefore, we examined the inhibitory effect of rapamycin (Rap), temsirolimus (Tem), and everolimus (Eve), on Ser2448-mTOR phosphorylation with a focus on rapalog-resistant TNBC cell lines HCC1806 and SUM149PT and rapalog-sensitive Hs578T TNBC cells. The rapalogs potently inhibited phosphorylation of mTOR in the sensitive Hs578T cells, but not or less effectively in the resistant HCC1806 and SUM149PT cells, respectively (Fig. 1c, d). These data suggest that mTOR kinase activity and its sustained phosphorylation render the TNBC cells resistant to rapalogs.

### Combinatorial drug screen identifies kinase inhibitors sensitizing TNBC cells to mTOR inhibition

**Fig. 2:**
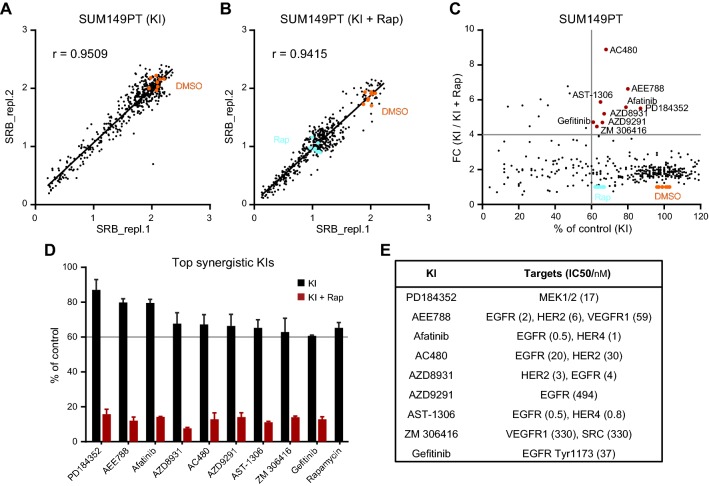
Identification of kinase inhibitors which sensitizes TNBC cells to rapamycin. **a**, **b** Pearson’s correlation coefficient r showing reproducibility of replica screen of 378 kinase inhibitors alone (KI, **a**) or combined with rapamycin (KI+ Rap, **b**). SUM149PT cells were treated for 4 days with 1 µM KI individuals alone or combined with 1 µM Rap. Orange dots, DMSO control. Cyan dots, Rap only. **c** Effect comparison of KI alone to KI combined with Rap on proliferation of SUM149PT cells. The percentage of proliferation (% of control) was relative to DMSO. The ratio of percentage of proliferation was shown as fold change (FC, KI versus KI + Rap). Top synergistic inhibitors were marked in red. **d** Selected inhibitors reducing 40% proliferation with FC > 4 (extracted from **c**, red dots) when combined with rapamycin. Error bars indicate screen replicates. **e** Kinase targets of the selected inhibitors and IC50 values of the inhibitors on corresponding targets (SelleckChem^®^)

Next, to identify kinase inhibitors synergizing with mTOR inhibition in rapalog refractory TNBC cells, we further performed a drug screen with rapamycin (at 1 µM) in combination with the 378 kinase inhibitors (also tested at 1 µM) in the resistant SUM149PT cells. Pearson’s correlation coefficient r displayed high reproducibility of two replicate screens for KI (*r* = 0.9509) and KI and rapamycin (KI + Rap, *r* = 0.9115), respectively (Fig. 2a, b). Comparison of KI + Rap combinatory effect to the single KI effect on proliferation inhibition uncovered 9 potent KIs (Fig. 2c), which significantly enhanced inhibitory effect of rapamycin on proliferation of SUM149PT cells (Fig. 2d). These included one MEK inhibitor PD184352 and 8 RTK inhibitors, AEE788, afatinib, AC480, AZD8931, AZD9291, AST-1306, ZM 306416, and gefitinib that are described to target single or multiple EGFR/HER2 and VEGFR RTKs (Fig. 2e). We also performed rapamycin combination screen in the resistant HCC1806 cells in parallel. As HCC1806 cells were responsive to EGFR inhibitors, only additive effects were observed (Suppl. Fig. S1c; Suppl. Fig. S2).

These data implicate that while the resistant SUM149PT cells poorly respond to inhibitors of EGFR or VEGFR and mTOR inhibitor rapamycin alone, concurrent blockage of upstream EGFR or VEGFR RTK activity or MEK signaling transduction, and downstream mTOR signaling could converge re-sensitization of TNBC cells.

### Multi-targeted RTK inhibitor AEE788 enhances proliferative inhibition and cell death in rapalog-resistant TNBC cells

**Fig. 3:**
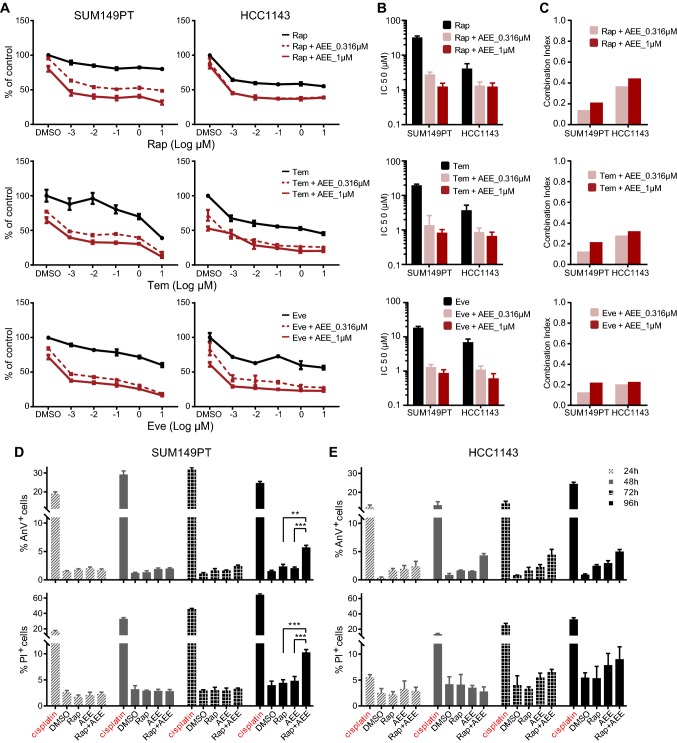
Synergistic effect of AEE788 and rapalogs on proliferative inhibition and cell death in rapalog-resistant TNBC cells. **a** Proliferative response of rapalog-resistant SUM149PT (left panel) and HCC1143 (right panel) TNBC cells, to rapalogs Rap, Tem, and Eve in concentration range alone or combined with 0.316 µM and 1 µM AEE788, respectively. **b** IC50 values (µM) of rapalogs in combination with AEE788, inducing 50% of proliferation inhibition in SUM149PT and HCC1143 cells. **c** Combination index (CI) of rapalog and AEE788 in SUM149PT and HCC1143 cells. CI < 1 indicates synergism. **d**, **e** Combinatorial effects of Rap and AEE788 on SUM149PT (left panel, **d**) and HCC1143 (right panel, **e**) cell death. Cells were subjected to Annexin V/Propidium Iodide (AnV/PI) apoptosis assays after treatment for 24, 48, 72, and 96 h. Cisplatin (100 µM) was used as positive control. (two-way ANOVA *p < 0.05, **p < 0.01, ***p < 0.001)

Next, we further validated the combinatorial effect of the most promising combinations on proliferative inhibition in the rapalog-resistant SUM149PT cells. We focused on the MEK inhibitor PD184352, EGFR inhibitor gefitinib, and the multi-targeted RTK inhibitor AEE788. Cells were treated with rapamycin in a concentration range alone or combined with different concentrations of PD184352, gefitinib, or AEE788. AEE788 synergized with rapamycin to inhibit SUM149PT cell proliferation in dose-dependent manner (Fig. 3a, top panel). In contrast, PD184352 and gefitinib (Suppl. Fig. S1a, b) displayed a more additive effect when combined with rapamycin. The synergistic effects of AEE788 and rapalogs, Rap, Tem, and Eve, were further confirmed in SUM149PT as well as another rapalog-resistant TNBC cell line HCC1143 (Fig. 3a). AEE788 significantly reduced the half-maximal inhibitory concentrations (IC50) of the rapalogs in both SUM149PT and HCC1143 cell lines (Fig. 3b). Combination index (CI) analysis detected the strong synergy of AEE788 and rapalogs, overall with CI values < 0.5 (Fig. 3c). To detect the combinatorial effects of AEE788 and rapamycin on TNBC cell death, we performed Annexin V/Propidium Iodide apoptosis assay in SUM149PT and HCC1143 cell lines. Besides proliferative inhibition, the combination significantly enhanced apoptosis and necrosis 96 h post treatment in SUM149PT cells (Fig. 3d), and enhanced the monotherapy-induced apoptosis in HCC1143 cells, albeit not statistically significant (Fig. 3e). Next, we evaluated the combinatorial effects on normal mammary cells MCF10A and renal cells RPTEC. Importantly, neither monotherapy nor combination significantly suppressed proliferation or induced cell death of MCF10A and RPTEC cells, suggesting that the combo effects might be cancer cell specific and less toxic in normal mammary and renal cells (Suppl. Fig. S3). Altogether, the KI combination not only inhibited TNBC cell proliferation, but also enhanced the monotherapy-induced apoptosis and necrosis, with minimal effects on normal mammary and renal cells.

### Co-treatment of rapamycin and AEE788 abolishes mTOR phosphorylation and sustains downregulation of ERK and AKT signaling in TNBC cells

**Fig. 4:**
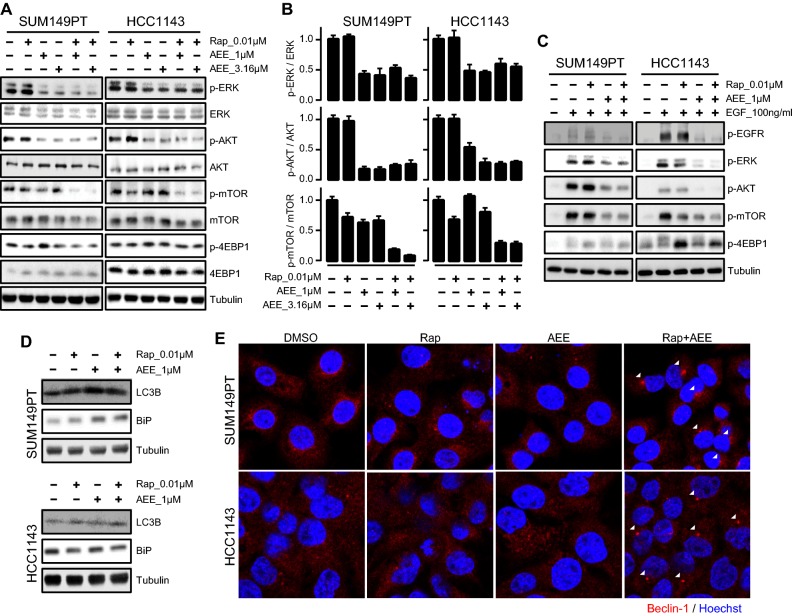
Combinatorial effect of AEE788 and rapamycin on mTOR signaling inhibition in rapalog-resistant TNBC cells. **a** Effects of AEE788 and Rap co-treatment on mTOR phosphorylation, upstream AKT and ERK and downstream 4EBP1 signaling in rapalog-resistant SUM149PT and HCC1143 cells. Cells were treated with 1–3.16 µM AEE788 and 0.01 µM Rap alone or combined as indicated for 4 h. **b** Quantification of phosphorylated ERK to total ERK (top row), phosphorylated AKT to total AKT (middle row), and phosphorylated mTOR to total mTOR (bottom row) in SUM149PT and HCC1143 treated with AEE788 and Rap alone or combined as indicated. **c** Effects of AEE788 and Rap co-treatment on EGF-stimulated signaling transduction in SUM149PT and HCC1143 cells. Cells were starved in serum-free medium overnight, pre-treated for 4 h with AEE788 and Rap alone or combined as indicated, followed by exposure to 100 ng/ml EGF for 5 min. 4EBP1 can be phosphorylated at several sites, as indicated by multiple bands. The bottom band is the unphosphorylated form of 4EBP1. **d** Effects of AEE788 and Rap co-treatment for 24 h on autophagy (LC3B) and ER stress (BiP). **e** Effects of AEE788 and Rap co-treatment on Beclin-1 accumulation. Cells were subjected to immunofluorescence assay 24 h post treatment. White arrows indicate the induction of Beclin-1

mTOR belongs to a complex network of regulatory feedback loops responsible for controlling upstream proliferative signaling pathways. The major upstream signaling in control of mTOR activity involves PI3K/AKT and MAPK/ERK, the two canonical pathways downstream of RTKs [7]. Resistance to mTOR inhibition in cancer has been linked to activation of upstream PI3K/AKT and MAPK/ERK signaling, following rapalog treatment [15]. Next, we investigated the synergistic effect of AEE788 and rapamycin on PI3K/AKT and MAPK signaling in TNBC cells. Treatment with AEE788 alone inhibited ERK and AKT phosphorylation in the resistant SUM149PT and HCC1143 cells (Fig. 4a). Single treatment with rapamycin slightly increased p-ERK in SUM149T and p-AKT in HCC1143 cells. The phosphorylation levels of mTOR and the target of mTOR, 4EBP1, were not affected by either AEE788 or rapamycin alone, further indicating the sustained mTOR signaling in the resistant TNBC cells. However, co-treatment of AEE788 and rapamycin almost completely abolished mTOR phosphorylation, while ERK and AKT phosphorylation remained inhibited (Fig. 4a, b). The synergistic effect of AEE788 and rapamycin on p-4EBP1 inhibition was marginal (Fig. 4a).

As AEE788 has been described as an EGFR/VEGFR dual RTK inhibitor, we further evaluated the co-treatment effect of AEE788 and rapamycin on EGFR RTK signaling activity in both resistant SUM149PT and HCC1143 cells upon EGF stimulation (Fig. 4c). To reach maximal activation of the EGFR signaling pathway, cells were first serum starved followed by EGF treatment. EGF caused the activation of the EGF receptor as evidenced by increased p-EGFR and downstream p-ERK and p-AKT. AEE788 effectively blocked EGF-stimulated phosphorylation of these components, in both SUM149PT and HCC1143 cells. It has been reported that 4EBP1 has multiple phosphorylation sites and an increase in 4EBP1 phosphorylation is accompanied by a decrease in its electrophoretic mobility [26–28]. EGF also effectively caused enhanced p-mTOR and p-4EBP1. Rapamycin could inhibit 4EBP1 phosphorylation by EGF in both cell lines but with no (SUM149PT) or limited (HCC1143) effects on mTOR activation; and, reversely, AEE788 could inhibit mTOR activation but with marginal effects on 4EBP1 phosphorylation. However, co-treatment with AEE788 and rapamycin particularly could shut down the EGF-mediated phosphorylation of mTOR and 4EBP1 signaling.

Several cellular processes have been linked to the immunogenicity of cell death, including autophagy and ER stress [29–32]. Given that mTOR is a key regulator of autophagy, we then tested the combination effect of autophagy in TNBC cells. Interestingly, the combination sustained the elevated LC3B level induced by monotherapy and demonstrated accumulated Beclin-1 expression 24 h post treatment in both SUM149PT and HCC1143 cells (Fig. 4d, e). The combination increased the expression level of BiP, a key regulator of ER stress, 24 h post treatment in SUM149PT cells, but not so much in HCC1143 cells (Fig. 4d), suggesting the involvement of immunogenic cell death-related events in TNBC cells by the combination treatment.

### Silencing of AEE788 targets enhances mTOR inhibition in TNBC cells

**Fig. 5:**
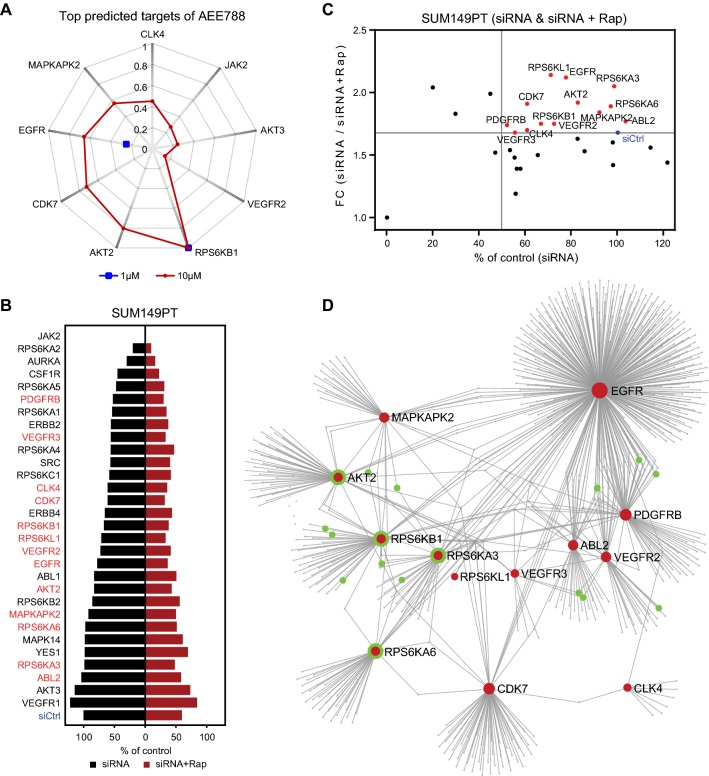
Validation of AEE788 potential targets synergizing with rapamycin in rapalog-resistant TNBC cells. **a** Radar chart displaying highly predicted targets of AEE788 in ChEMBL_23 database with 1 µM (blue) and 10 µM (red) as cutoff. **b** Screen of AEE788 targets with siRNA alone (siRNA) or in combination with 0.01 µM rapamycin (siRNA + Rap) in SUM149PT cells. siCtrl, siRNA control. **c** siRNA silencing effect of AEE788 targets synergizing with rapamycin. The ratio of percentages of proliferation was shown as FC (siRNA versus siRNA + Rap). Targets with silencing effect over siCtrl (FC > 1.7, blue) were marked in red. **d** Protein–protein interactions of AEE788 targets (red) by NetworkAnalyst. Proteins interacting with mTOR signaling pathway were marked in green

Although AEE788 has been described as an inhibitor targeting multiple RTKs, we wondered whether the effect of AEE788 could be related to unanticipated polypharmacology, thus impacting through additional mechanisms of mTOR signaling. We used a cheminformatics approach to predict candidate alternative kinase targets of AEE788. ChEMBL is an open large-scale bioactivity database that contains comprehensive target inhibition information of thousands of drug-like molecules, including kinase inhibitor activity, allowing well-informed prediction of structure-based alternative kinase target prediction [33, 34]. We firstly performed ligand-based target prediction for AEE788. With 1 µM and 10 µM activity cutoffs, 9 kinases showed high prediction scores and as such putative targets of AEE788, including RPS6KB1, AKT2, CDK7, EGFR, MAPKAPK2, CLK4, JAK2, AKT3, and VEGFR2 (Fig. 5a). To refine the scale of target list, we selected kinases showing prediction score greater than 0.1 and kinases with an IC50 of AEE788 smaller than 1 µM according to the publically available data [35]. As a result, 30 putative kinase targets were selected. To validate the potential contribution of these kinases in the interaction with rapamycin, we performed a targeted rapamycin and siRNA synthetic lethal screen in SUM149PT cells (Fig. 5b). The synthetic lethal screen revealed 13 candidate targets (Fig. 5c). We anticipated that these validated targets would take part in connected signaling networks and, therefore, would all individually impact on the rapamycin sensitivity. Indeed, protein–protein interaction network analysis revealed a close interaction of the various putative kinase targets of AEE788 (Fig. 5d; Suppl. Table S2). Interestingly, the well-known mTOR target RPS6KB1 as well as other RPS6K family members RPS6KA3, RPS6KA6, and RPS6KL1 were mapped in the network, supporting the synergistic drug interaction of AEE788 with rapamycin on mTOR signaling. In addition, ABL2 and PDGFRB were predicted and validated as potential targets involved in rapamycin synergy. Of relevance, rapalog-resistant TNBC cell lines SUM149PT, HCC1143, SUM159PT, and HCC38 poorly responded to inhibitors targeting the verified targets of AEE788, including EGFR, VEGFR, PDGFR, ABL, and S6K (Suppl. Fig. S2). Taken together, the above data suggest that AEE788 synergizes with rapamycin in suppressing TNBC cell proliferation by targeting several EGFR, VEGFR, PDGFR, ABL, and different S6K kinases that are all connected to mTOR signaling.

### AEE788 abolished rapalog-upregulated cyclin D1 expression in TNBC cells

**Fig. 6:**
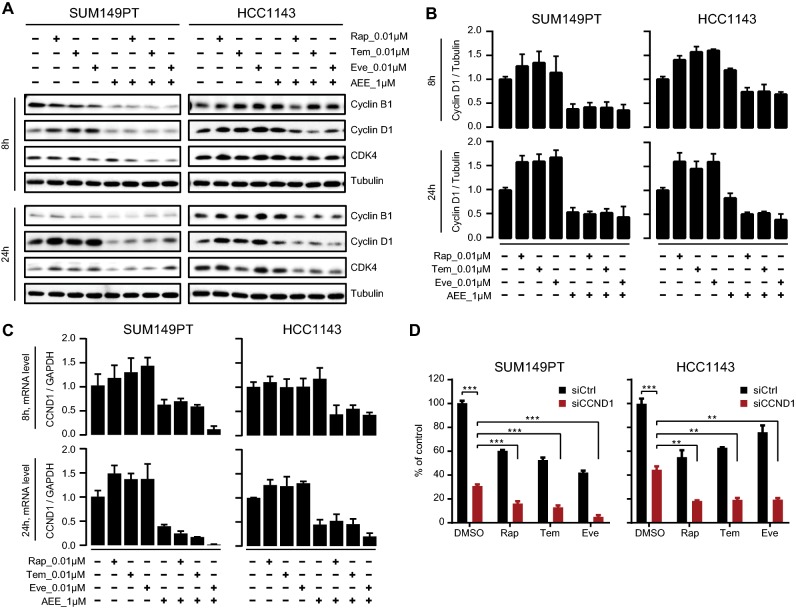
Co-treatment with AEE788 prevents rapalog-induced cyclin D1 upregulation in resistant TNBC cells. **a** Combinatorial effects of AEE788 and rapalogs (Rap, Tem, and Eve) on expression of cell cycle regulatory proteins in SUM149PT and HCC1143 cells. Cells were treated with 1 µM AEE788 and 0.01 µM rapalogs (Rap, Tem, and Eve) alone or combined as indicated, for 8 h and 24 h, respectively. **b** Cyclin D1 protein expression levels relative to tubulin in SUM149PT and HCC1143 cells treated with AEE788 and rapalogs alone or combined as indicated. **c** Cyclin D1 mRNA expression level relative to GAPDH in SUM149PT and HCC1143 cells treated with AEE788 and rapalogs alone or combined as indicated. **d** Effect of CCND1 siRNA silencing (siCCND1) on proliferation inhibition of SUM149PT and HCC1143 cells treated with 0.01 µM rapalogs or DMSO control (two-way ANOVA *p < 0.05, **p < 0.01, ***p < 0.001). siCtrl, siRNA control. Significant effect of rapalogs treatment alone on proliferative inhibition was observed (**p < 0.01)

Finally, we looked into the mechanism how AEE788 and rapalogs impact on cell proliferation. Inhibition of mTOR by rapamycin blocks cell cycle progression, and cell proliferation has been linked to disruption of the cyclin-dependent kinase 4 (CDK4)-cyclin D1 complex [36]. Therefore, we next addressed the role of cyclin D1 in the synergistic effect of AEE788 and rapalogs on proliferation of rapalog-resistant SUM149PT and HCC1143 TNBC cell lines. Unexpectedly, we observed that rapalogs Rap, Tem, and Eve did not suppress but upregulated cyclin D1 protein expression and mRNA levels of CCND1 (the gene encoding cyclin D1) in SUM149PT and HCC1143 TNBC cells after short-term (8 h) and long-term (24 h) treatments (Fig. 6a–c). This suggests a positive-feedback loop activation upon rapalog treatment, thereby counteracting the anti-proliferative effect of rapalogs. Rapalogs slightly increased CDK4 levels, but did not affect cyclin B1 expression in the TNBC cells (Fig. 6a). When co-treated with AEE788, the rapalog-induced cyclin D1 upregulation was blocked at both the mRNA and protein levels (Fig. 6a–c). Moreover, co-treatment of AEE788 and rapalogs led to downregulation of cyclin B1 and CDK4 expression in both TNBC cell lines (Fig. 6a). These results suggested that AEE788 synergized with rapalog to abrogate cyclin D1 upregulation thereby inhibiting cell proliferation. Next, we silenced cyclin D1 by siRNA-based CCND1 knockdown in SUM149PT and HCC1143 cells, in combination with rapalogs Rap, Tem, and Eve, respectively. While silencing cyclin D1 alone considerably impaired SUM149PT and HCC1143 cell proliferation, this inhibitory effect was significantly enhanced when combined with rapalogs Rap, Tem, or Eve (Fig. 6d). In support of a role of CDK4/cyclin D1 in the resistant phenotype of rapamycin, an enhanced inhibition on proliferation was observed in SUM149PT and HCC1143 cells when co-treated with rapamycin and selective CDK4/6 inhibitor palbociclib or LY2835219 (Suppl. Fig. S3), albeit not as effective as AEE788.

## Discussion

mTOR acts as a central regulator of multiple signaling networks in control of cell growth, proliferation, and survival [7, 37]. mTOR signaling is frequently upregulated in malignant tumors, including TNBC, highlighting the potential of mTOR kinase targeted therapy in cancer modulation [7–9, 12]. However, patients with TNBC often experience mTOR targeting failure due to acquired resistance and activation of bypass surviving pathways [13, 15, 38]. Our drug combination screen revealed that co-treatment with AEE788, a multiple RTK-targeted inhibitor, restores the sensitivity of TNBC cells towards the clinically applied mTOR inhibitors (rapamycin, temsirolimus, and everolimus). The effect of AEE788 is likely due to polypharmacology to shut down the crosstalk among receptors as well as mTOR pathway within signaling networks in the resistant scenario. The combination of targeted agents profoundly improves therapeutic efficacy and overcomes resistance that might develop under single-agent therapy.

mTOR inhibition can relieve distinct negative feedback loops that normally serve to attenuate upstream RTKs, PI3K, and MAPK signaling, leading to rapalog resistance [39]. As such, mTOR inhibition alone is not sufficient to overcome the entire oncogenic program propagated from the alternate proliferative signaling pathways. By exploiting a high-throughput kinase drug combination screen, our study has identified the effective kinase inhibitor, AEE788, that can block compensatory mechanisms conferring aberrant cell cycle progression upon rapalog treatment. The repression of EGFR/VEGFR- and mTOR-related pathways in concert seemingly reverts processes predominantly responsible for uncontrolled TNBC tumor proliferation. Our results are in line with the above observations that co-inhibition of upstream RTKs (such as EGFR, VEGFR, PDGFR, and IGF1R), PI3K and MAPK signaling transduction, and mTOR signaling elicited enhanced therapeutic efficacy in various cancer types.

Sustained mTOR signaling drives resistance to targeted therapeutics in cancer treatment [38]. In TNBC tumor cells, mTOR signaling is frequently upregulated [40]. We demonstrated that while rapalogs alone were insufficient to inhibit the sustained mTOR signaling in resistant TNBC cells, co-treatment of rapalogs with the multi-targeted RTK inhibitor AEE788 synergistically blocked mTOR phosphorylation in SUM149PT and HCC1143 cells. Interestingly, SUM149PT cells have been characterized for the constitutively activated EGFR via a self-sustaining amphiregulin autocrine loop, and subsequently, altered receptor signaling and gene expression [41, 42]. As a further validation, AEE788 and rapamycin treatment blocked EGF-mediated EGFR downstream signaling in both SUM149PT and HCC1143. We recognize that these experiments were performed under short-term EGF treatment conditions that may not fully reflect the normal TNBC cell signaling. Therefore, we cannot exclude that under more physiological conditions in the tumor microenvironment other distinct RTK signaling cascades are more prominent in the modulation of RTK signaling and cancer progression.

A mechanism of resistance to mTOR inhibition in cancer is the rapalog-mediated activation of upstream PI3K/AKT and MAPK/ERK signaling [15]. Co-treatment with AEE788 and rapamycin maintained the inhibitory effect on AKT and ERK signaling in TNBC cells. These data suggest that AEE788 and rapamycin synergistically inhibit the sustained mTOR activity in TNBC cells, thus blocking mTOR’s potential feedback loop on activation of alternative ERK and AKT proliferative signaling pathways.

Polypharmacology, the action of drugs against multiple targets [17], is commonly observed in drug development including the effective marketed kinase inhibitors [43]. Our ChEMBL-based cheminformatics analysis demonstrated that AEE788 is a kinase inhibitor that likely targets several kinases; this is consistent with other reports using protein kinase assays [19, 35]. Complementary to a recent kinobeads study on target landscape of clinical kinase drugs [43], our cheminformatics approach presented that the multi-targeted RTK inhibitor AEE788 likely interacts with EGFR, VEGFR, ABL2, PDGFRB, and several mTOR signaling pathway components, including AKT and S6K family members. Subsequent siRNA-based knockdown of these various kinases, e.g., RTKs (EGFR, VEGFR2/3, and PDGFRB), AKTs (AKT2 and AKT3), RPS6Ks (RPS6KA3, RPS6KA6, RPS6KB1, and RPS6KL1), MAPKAPK2, ABL2, and CDK7, sensitized rapalog-resistant TNBC cells to rapamycin. Several reports have demonstrated the synergistic effects of targeting EGFR or MEK on anti-mTOR therapies in TNBC [20, 44–46]. However, our study demonstrated that simultaneous use of EGFRi gefitinib or MEKi PD184352 only exerts additive effects on rapamycin-mediated proliferative inhibition, suggesting that AEE788-rapalog synergy results presumably from multi-targeted kinase inhibition. These data support the anticipated polypharmacology of AEE788 as the mode-of-action of the synergy with rapalogs. Further studies are required to determine the detailed kinome target landscape of AEE788 in TNBC.

mTOR pathway regulates cell growth through its downstream effectors, such as 4EBP1 and RPS6KB1 [7, 37]. Another primary way that mTOR confers its regulatory effects on cell proliferation is to upregulate expression of the cell cycle regulator cyclin D1 [47]. CCND1, the cyclin D1 encoding gene, is frequently amplified in breast cancer, and depletion of cyclin D1 suppresses breast cancer progression [48, 49]. In response to mTOR inhibition, however, cyclin D1 is elevated by everolimus in various types of cancer [21, 22]. Consistently, we found that treatment with rapalogs (rapamycin, temsirolimus, and everolimus) commonly upregulated cyclin D1 in rapalog-resistant TNBC cells, indicating an alternative activation of cyclin D1 proliferative signaling pathway after mTOR inhibition. Considering that cyclin D1 was lost in the presence of the AEE788-rapalog combination, AEE788 seems to compensate the undesired effects of rapalog, further highlighting the therapeutic advantage of the drug combination. Interestingly, while we discovered the AEE788-rapamycin interaction through a wider screening effort in TNBC cells, our findings were further supported by the observations on the synergistic effects of AEE788-everolimus combination in prostate, germ, and renal tumor cell lines [19, 21, 22]. Moreover, a xenograft-bearing mice study also documented the beneficial action of AEE788-everolimus combination in glioblastoma tumor regression [50]. However, these studies did not further the mode-of-action of AEE788. Since AEE788 is recognized as a multiple targeting kinase inhibitor, their observations were limited to EGFR/VEGFR, lacking the notion on other potentially targeted candidate kinases. Our study, for the first time, revealed the synergy on rapalogs treatment in TNBCs and its underlying polypharmacology by utilizing integrated systematic screen and cheminformatics approach. Moreover, either genetic or pharmacological ablation of cyclin D1 significantly enhanced mTOR-inhibition-mediated proliferative inhibition. This is concordant with the recent reports on the synergistic anti-cancer activity of combined CDK4/6 and mTOR targeting [51–53].

In conclusion, our work supports that polypharmacology to target multiple kinase targets in combination with rapalog treatment may offer a distinct combinatorial benefit to TNBC patients that are otherwise resistant to mTOR-targeted therapeutics.

## Electronic supplementary material

Below is the link to the electronic supplementary material.

Supplementary material 1 (PDF 17805 kb) **Suppl. Fig. S1.** Effects of co-treatment with PD184352, gefitinib or AEE788 on rapamycin-mediated proliferative inhibition in TNBC cells. SUM149PT (**a**, **b**) and HCC1806 (**c**) cells were treated with Rap in dose range alone or combined with PD184352 (PD), gefitinib (Gef) or AEE788 (AEE) at indicated concentrations for 4 days, followed by SRB proliferation assay. **Suppl. Fig. S2.** Proliferation response of rapalog-resistant TNBC cell lines towards VEGFR, EGFR, PDGFR, ABL and S6K inhibitors. TNBC cells were treated with KI at 1 µM for 4 days, followed by SRB proliferation assay. Strong inhibitory effect on proliferation was indicated in green and weak in red. **Suppl. Fig. S3.** Combinatorial effect of rapamycin and AEE788 on proliferation and cell death of MCF10A (**a**) and RPTEC (**b**) cells. Proliferative response (upper panel, SRB absorbance), early apoptosis (middle panel, AnV^+^) and late apoptosis/necrosis (bottom panel, PI^+^) of MCF10A and RPTEC cells, to Rap alone or combined with AEE788 respectively. Cisplatin (100 µM) was used as positive control. **Suppl. Fig. S4.** Combinatorial effect of rapamycin and inhibitors targeting CDK4/6-Cyclin D1 complexes on proliferation of rapalog-resistant SUM149PT (**a**) and HCC1143 (**b**) TNBC cells. Cells were treated Rap alone, or in combination with selective CDK4/6 inhibitor palbociclib or LY2835219 at 0.01 µM for 4 days (two-way ANOVA *p < 0.05, **p < 0.01, ***p < 0.001).

Supplementary material 2 (XLSX 53 kb) **Suppl. Table S1.** Molecular subtypes of TNBC cell lines. **Suppl. Table S2.** Node table in protein-protein interactions of AEE788 potential targets.

Supplementary material 3 (DOC 59 kb) Supplementary material and methods.

## Abbreviations

BL: Basal-like
CDK4: Cyclin-dependent kinase 4
CI: Combination index
EGF: Human epidermal growth factor
FC: Fold change
IC50: Half-maximal inhibitory concentration
IM: Immunomodulatory
KI: Kinase inhibitor
LAR: Luminal androgen receptor-like
MSL: Mesenchymal stem-like
mTOR: The mammalian target of rapamycin
RTK: Receptor tyrosine kinase
SRB: Sulforhodamine B
TNBC: Triple-negative breast cancer
