# Supplementary material for: Multi-targeted kinase inhibition alleviates mTOR inhibitor resistance in triple-negative breast cancer
Source: Breast Cancer Res Treat. 2019 Aug 6;178(2):263–74. doi: 10.1007/s10549-019-05380-z (PMC6797661; doi:10.1007/s10549-019-05380-z)
Supplement: Supplementary file 1 — Supplementary material 1 (PDF 17805 kb) Suppl. Fig. S1. Effects of co-treatment with PD184352, gefitinib or AEE788 on rapamycin-mediated proliferative inhibition in TNBC cells. SUM149PT (a, b) and HCC1806 (c) cells were treated with Rap in dose range alone or combined with PD184352 (PD), gefitinib (Gef) or AEE788 (AEE) at indicated concentrations for 4 days, followed by SRB proliferation assay. Suppl. Fig. S2. Proliferation response of rapalog-resistant TNBC cell lines towards VEGFR, EGFR, PDGFR, ABL and S6K inhibitors. TNBC cells were treated with KI at 1 µM for 4 days, followed by SRB proliferation assay. Strong inhibitory effect on proliferation was indicated in green and weak in red. Suppl. Fig. S3. Combinatorial effect of rapamycin and AEE788 on proliferation and cell death of MCF10A (a) and RPTEC (b) cells. Proliferative response (upper panel, SRB absorbance), early apoptosis (middle panel, AnV+) and late apoptosis/necrosis (bottom panel, PI+) of MCF10A and RPTEC cells, to Rap alone or combined with AEE788 respectively. Cisplatin (100 µM) was used as positive control. Suppl. Fig. S4. Combinatorial effect of rapamycin and inhibitors targeting CDK4/6-Cyclin D1 complexes on proliferation of rapalog-resistant SUM149PT (a) and HCC1143 (b) TNBC cells. Cells were treated Rap alone, or in combination with selective CDK4/6 inhibitor palbociclib or LY2835219 at 0.01 µM for 4 days (two-way ANOVA *p < 0.05, **p < 0.01, ***p < 0.001). [file 10549_2019_5380_MOESM1_ESM.pdf]

Multi-targeted kinase inhibition alleviates mTOR inhibitor resistance in triple negative breast cancer

Jichao He[1], Ronan P. McLaughlin[1], Vera van der Noord[1], John A. Foekens[2], John W. M. Martens[2], Gerard van Westen[1], Yinghui Zhang[1], Bob van de Water[1,3]

[1]Division of Drug Discovery and Safety, Leiden Academic Centre for Drug Research, Leiden University, 2300 RA Leiden, the Netherlands
[2]Department of Medical Oncology and Cancer Genomic Netherlands, Erasmus MC Cancer Institute, Erasmus Medical Centre, 3000 CA Rotterdam, the Netherlands.

[3]Correspondence: Bob van de Water, Division of Drug Discovery and Safety, Leiden Academic Centre for Drug Research, Leiden University, 2300 RA Leiden, the Netherlands.
Email: b.water@lacdr.leidenuniv.nl; phone: +31-71--5276223

ELECTRONIC SUPPLEMENTAL MATERIAL

Figures S1-4

Supplementary Figure Legends

Suppl. Fig. S1

Effects of co-treatment with PD184352, gefitinib or AEE788 on rapamycin-mediated proliferative inhibition in TNBC cells. SUM149PT **(a, b)** and HCC1806 **(c)** cells were treated with Rap in dose range alone or combined with PD184352 (PD), gefitinib (Gef) or AEE788 (AEE) at indicated concentrations for 4 days, followed by SRB proliferation assay.

Suppl. Fig. S2

Proliferation response of rapalog-resistant TNBC cell lines towards VEGFR, EGFR, PDGFR, ABL and S6K inhibitors. TNBC cells were treated with KI at 1 µM for 4 days, followed by SRB proliferation assay. Strong inhibitory effect on proliferation was indicated in green and weak in red.

Suppl. Fig. S3

Combinatorial effect of rapamycin and AEE788 on proliferation and cell death of MCF10A **(a)** and RPTEC **(b)** cells. Proliferative response (upper panel, SRB absorbance), early apoptosis (middle panel, AnV^+) and late apoptosis/necrosis (bottom panel, PI^+) of MCF10A and RPTEC cells, to Rap alone or combined with AEE788 respectively. Cisplatin (100 µM) was used as positive control.

Suppl. Fig. S4

Combinatorial effect of rapamycin and inhibitors targeting CDK4/6-Cyclin D1 complexes on proliferation of rapalog-resistant SUM149PT **(a)** and HCC1143 **(b)** TNBC cells. Cells were treated Rap alone, or in combination with selective CDK4/6 inhibitor palbociclib or LY2835219 at 0.01 µM for 4 days (two- way ANOVA * $p < 0.05$, ** $p < 0.01$, *** $p < 0.001$).

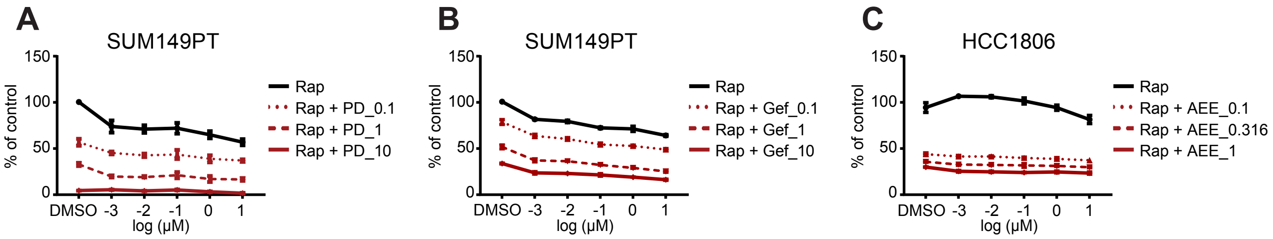

He et al Suppl. Fig. S1

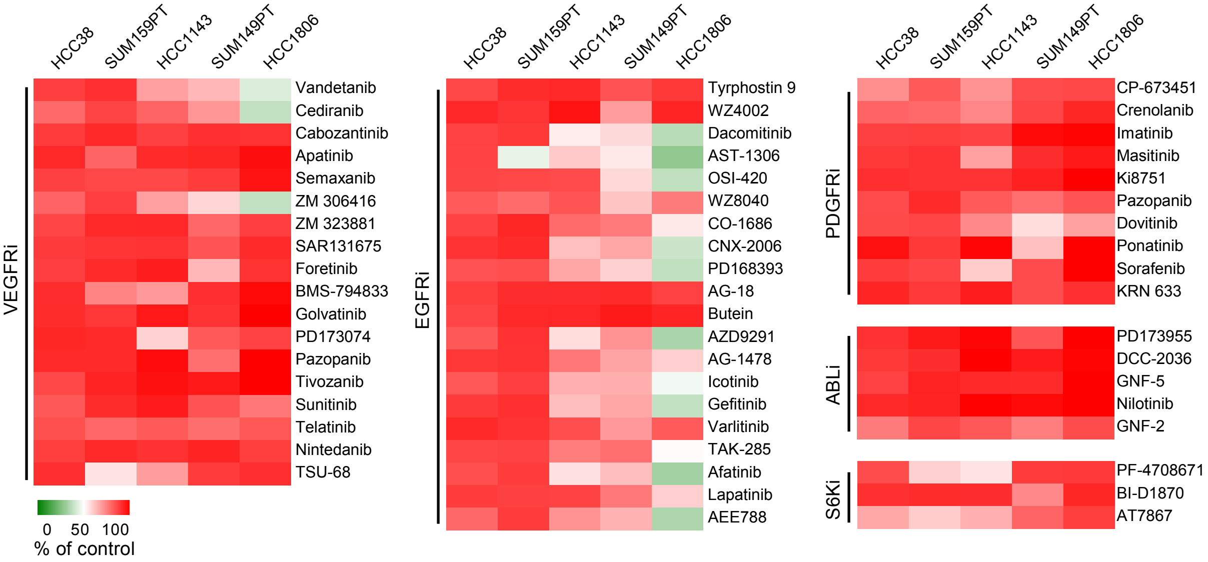

He et al Suppl. Fig. S2

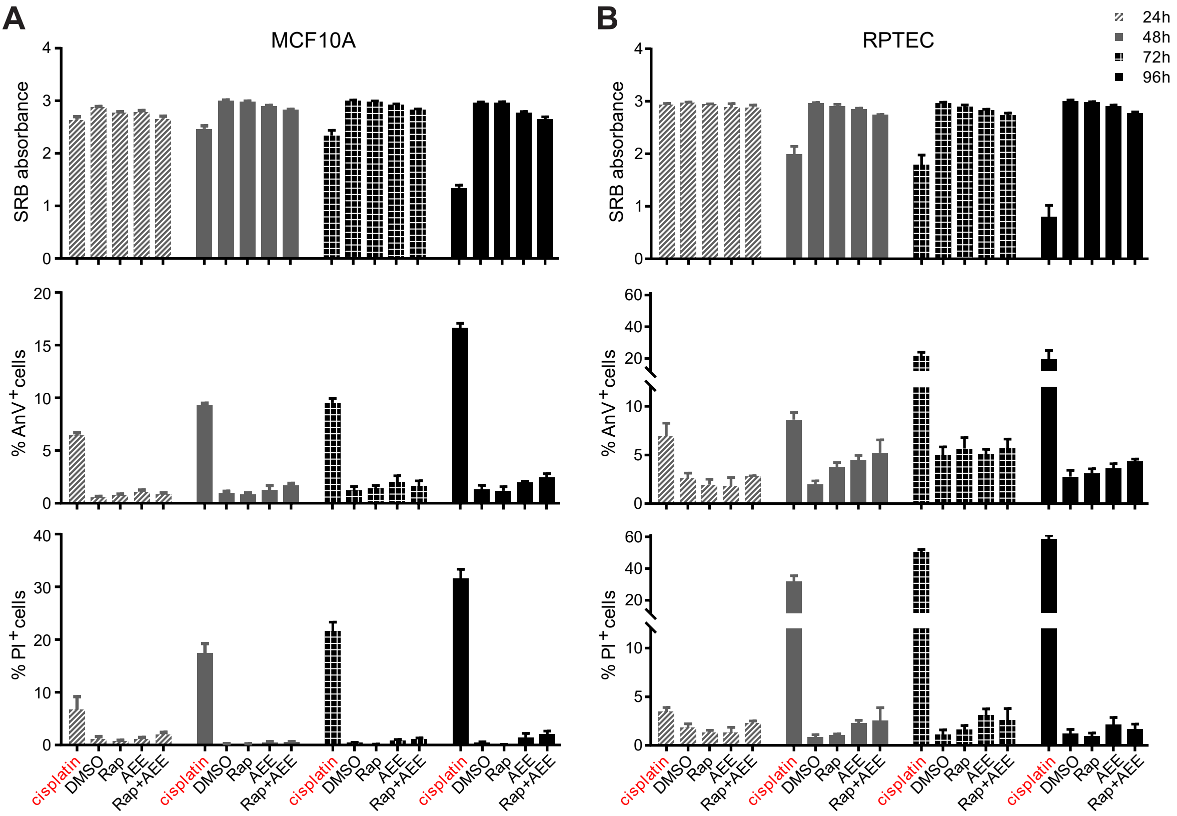

He et al Suppl. Fig. S3

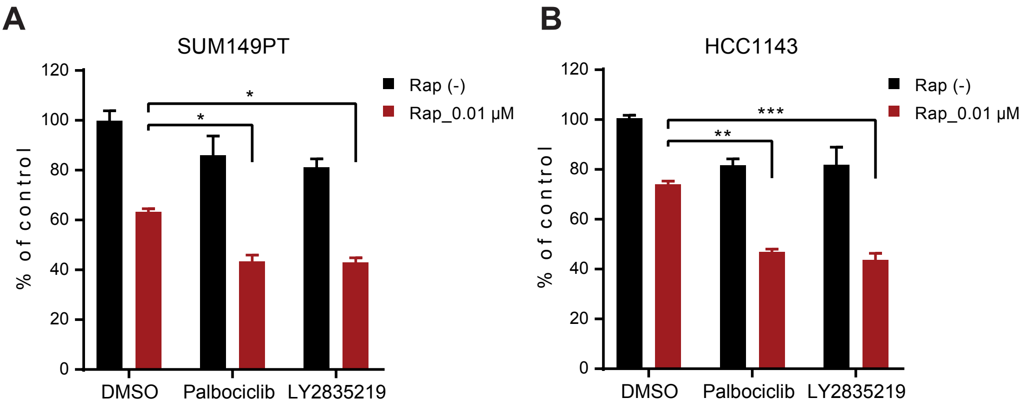

He et al Suppl. Fig. S4
