# Supplementary material for: Multi-targeted kinase inhibition alleviates mTOR inhibitor resistance in triple-negative breast cancer
Source: Breast Cancer Res Treat. 2019 Aug 6;178(2):263–74. doi: 10.1007/s10549-019-05380-z (PMC6797661; doi:10.1007/s10549-019-05380-z)
Supplement: Supplementary file 3 — Supplementary material 3 (DOC 59 kb) Supplementary material and methods. [file 10549_2019_5380_MOESM3_ESM.doc]

**Multi-targeted kinase inhibition alleviates mTOR inhibitor resistance in triple negative breast cancer**

Jichao He^1^, Ronan P. McLaughlin^1^, Vera van der Noord^1^, John A. Foekens^2^, John W. M. Martens^2^, Gerard van Westen^1^, Yinghui Zhang^1^, Bob van de Water^1,3^

^1^Division of Drug Discovery and Safety, Leiden Academic Centre for Drug Research, Leiden University, 2300 RA Leiden, the Netherlands

^2^Department of Medical Oncology and Cancer Genomic Netherlands, Erasmus MC Cancer Institute, Erasmus Medical Centre, 3000 CA Rotterdam, the Netherlands.

^3^Correspondence: Bob van de Water, Division of Drug Discovery and Safety, Leiden Academic Centre for Drug Research, Leiden University, 2300 RA Leiden, the Netherlands.

Email: [b.water@lacdr.leidenuniv.nl](mailto:b.water@lacdr.leidenuniv.nl); phone: +31-71--5276223

**ELECTRONIC SUPPLEMENTAL MATERIAL**

**Supplementary material and methods**

**Supplementary material and methods**

**Cell culture**

TNBC cell lines used were representative for different TNBC subtypes, including basal-like 1 (BL1) HCC38, HCC1143, HCC1937 and MDA-MB-468, basal-like 2 (BL2) HCC70, HCC1806 and SUM149PT, mesenchymal (M) BT549, mesenchymal stem-like (MSL) Hs578T, MDA-MB-231, MDA-MB-436 and SUM159PT, luminal androgen receptor (LAR) MDA-MB-453 and SUM185PE, and unclassified BT20, SKBR7, SUM52PE, SUM229PE and SUM1315MO2. All human TNBC cell lines were cultured in RPMI-1640 medium supplemented with 10% fetal bovine serum, 25 U/mL penicillin and 25 µg/mL streptomycin in a humidified incubator at 37°C with 5% CO_2_. Normal breast cell line MCF10A was kindly provided by Prof. dr. Peter ten Dijke (LUMC, Leiden, the Netherlands) and maintained in DMEM/F12 (Gibco) supplemented with 5% horse serum (Gibco), 20ng/ml epidermal growth factor (EGF) (Upstate), 100ng/ml cholera toxin (Calbiochem), 0.5 g/ml hydrocortisone (Sigma), 10 g/ml insulin (Sigma), 100U/ml penicillin and 50 g/ml streptomycin (Gibco). Kidney cell line RPTEC was cultured in DMEM/F12 (Gibco), containing a final concentration of 5 mM glucose, and supplemented with 2 mM Glutamax, 10 ng/ml epidermal growth factor, 36 ng/ml hydrocortisone, 5 µg/ml insulin, 5 µg/ml transferrin, 5 ng/ml selenium, and 100 U/ml penicillin and 100 µg/ml streptomycin, and grown for over 10 days after confluency to demonstrate a differentiated phenotype.

**Reagents and antibodies**

The library of 378-kinase inhibitors (L1200), rapamycin, temsirolimus, everolimus, AEE788, gefitinib, PD184352, palbociclib, and LY2835219 inhibitors were purchased from SelleckChem (Huissen, Netherlands). The phospho(Ser473)-AKT (9271), phospho(Thr202/Tyr204)-p44/42 MAPK (ERK1/2, 9101), phospho(Ser2448)-mTOR (5536S), phospho(Thr37/46)-4EBP1 (2855), phospho((Tyr1148))-EGFR (4404), 4EBP1 (8594), Cyclin B1 (4135), mTOR (4517), Beclin-1 (3738), AKT (9272) and p44/42 MAPK (ERK1/2, 4695) antibodies were from Cell Signaling (Bioké, Leiden, Netherlands). Cyclin D1 (sc-20044) and CDK4 (sc-601) antibodies were from Santa Cruz (CA, USA), The antibody against tubulin (T-9026) and human epidermal growth factor (EGF, E9644) were from Sigma Aldrich (Zwijndrecht, The Netherlands). The antibody against GRP78/BiP (610978) was from BD Biosciences (NJ, USA). The LC3B (NB100-2220) antibody was from Novus biologics (Colorado, USA).

**siRNA transfection**

To silence target genes, 50 nM siGENOME Human SMARTpool siRNA mix (GE Dharmacon, Lafayette, CO, USA) was transfected into cells by transfection reagent INTERFERin (Polyplus-Transfection SA, Illkirch-Graffenstaden, France) according to the manufacturer’s instructions. A pool of 720 kinase siRNAs at stock concentration of 1 µM, which has negligible effect on gene expression, was taken as control. The medium was refreshed 24 h post-transfection and transfected cells were used for experiments 48 h post-transfection.

**SRB proliferation assay**

A sulforhodamine B (SRB) colorimetric assay was used to measure total amount of proteins indicative of cell proliferation. Briefly, 4-day post-treatment, cells in 96-well plates were fixed with 30 µL 50% trichloroacetic acid for 1 hour at 4°C, followed by gently washing with tap water. Fixed and air-dried cells were stained with 60 μL of 0.4% SRB (dissolved in 1% acetic acid) at room temperature for 2 h. Unbound SRB was washed away with 1% acetic acid. The protein-bound SRB was solubilized in 10 mM unbuffered Tris base solution at room temperature for 2 h on a plate shaker and measured for its absorbance at 540 nm with an Infinite M1000 microplate reader (Tecan, Giessen, the Netherlands).

**Annexin V/Propidium Iodide apoptosis assay**

To detect apoptosis, a live cell imaging of Annexin V-Alexa633/Propidium Iodide (AnV/PI) labeling was performed in real time. Cells were treated as indicated and labeled with AnV (250 ng/ml) that conjugates to phosphatidyl serine on the membranes of apoptotic cells, and PI (100 nM) that intercalates with DNA in apoptotic or necrotic cells. At the time points of 24, 48, 72 and 96 h, the AnV and PI in-taken cells were captured with a Nikon Eclipse Ti confocal microscope. Simultaneously, the nuclei of live cells were stained with DNA dye Hoechst 33342 (200 ng/ml) and imaged for cell density. Quantitative image analysis was performed with CellProfiler (v2.1.1). AnV and PI apoptosis fraction was calculated by normalization of AnV and PI positive cells to the total cell number.

**Immunofluorescence assay**

SUM149PT and HCC1143 cells were fixed 24 h after treatment with ice cold methanol for 15 minutes, and were subsequently rinsed 3 times for 5 minutes with PBS. Afterwards, the cells were incubated with blocking solution (10% normal goat serum, 0.3% Triton-100 in PBS) for 1 h, rinsed 3 times for 5 min with PBS, followed by overnight incubation with primary antibody (1:300), washing, and 1 h incubation with second antibody. Nuclei stainig with Hoechst 33342 was performed as a final step together with the rinsing steps. The antibodies were diluted in antibody staining solution (1% BSA, 0.3% Triton-100 in PBS). All images were taken with confocal microscope Eclipse Ti-E from Nikon.

**Synergy assessment**

Combination Index (CI) was used to define synergism (CI < 1), additive effect (CI = 1) and antagonism (CI > 1) of combination drug treatment. The concentration of the single drug that inhibits 50% of cell proliferation (IC_50_) was determined by fitting the dose-response curve using GraphPad Prism 7.0 software. The CI was calculated using the formula “CI = C_A, 50_/IC_50, A_ + C_B, 50_/IC_50, B_”, where C_A, 50_ and C_B, 50_ are the concentration of drug A and B used in combination to achieve 50% drug effect.

**Western Blotting**

Cells were seeded in 6-well plates at the appropriate density. For stimulation/starvation assays, medium was refreshed with serum-free medium (SFM) the following day and cells were starved overnight. Thereafter, cells were pre-treated with drug solutions for 4 hours, then stimulated with 100 ng/ml EGF (Sigma; E9644) for 5 minutes in SFM. Cells were lysed with RIPA buffer containing 1% protease/phosphatase inhibitor cocktail (Sigma-Aldrich, P8340). Proteins were resolved by SDS-PAGE and transferred to polyvinylidine difluoride membranes. Membranes were blocked in 5% BSA in Tris-buffered saline with 0.05% Tween-20 (TBS-T), followed by overnight incubation with primary antibodies, washing, and 1 h incubation with HRP-conjugated secondary antibodies. Chemiluminescence was generated in the presence of HRP substrate and detected with an Amersham Imager 600 (GE Healthcare Life Sciences, Eindhoven, the Netherlands). Whenever relevant, the intensity of protein band was quantified using ImageJ software.

**Real time PCR (qPCR) assay**

RNA was isolated from TNBC cells using RNeasy (Qiagen). cDNA was generated from 400 ng total RNA, using RNeasy Plus Kit from Qiagen. Real-time qPCR was performed in triplicate, using the SYBRGreen PCR MasterMix (Applied Biosystems) on a 7900HT fast real-time PCR system (Applied Biosystems). The primer sequences used were: forward *ATCAAGTGTGACCCGGACTG*, reverse *CTTGGGGTCCATGTTCTGCT* (human CCND1); forward *CTGGTAAAGTGGATATTGTTGCCAT*, reverse *TGGAATCATATTGGAACATGTAAACC* (human GAPDH). Relative mRNA levels after correction for GAPDH control mRNA were expressed using the 2^-ΔΔCT^ method.

**Putative target prediction, validation and network analysis**

Candidate kinase targets of AEE788 were predicted by ligand-based target prediction model in ChEMBL database (version 23) and validated by siRNA knockdown plus rapamycin treatment. Bioactivity data for single protein targets in ChEMBL was used to train and validate two Naive Bayesian multi-label classifier models (at 1 µM and 10 µM bioactivity cutoffs respectively). Specifically, the model learns what sub-structural features of ligands correlate with activity against a certain target and assigns a score to each of these features. Bioactivity data was filtered for the presence of a pChEMBL value and only data with confidence score 9 was used. The model sums the individual feature scores for all the targets and comes up with a sorted list of likely targets with the highest scores. Validated gene targets showing higher FC (fold change) than control siRNA were taken as input to perform protein-protein interaction analysis in NetworkAnalyst (http://www.networkanalyst.ca/NetworkAnalyst/faces/home.xhtml) using IMEx Interactome database. KEGG pathway database was used to generate gene network.

**Statistical analysis**

Kinase inhibitor (KI) library screen data were analyzed using an unbiased sample-based analysis with the formula “Z score = (individual KI sample - mean of all KI samples)/standard deviation of all KI samples”. The effect of individual KIs on cell proliferation inhibition was considered significant when their Z score < -1.5. Pearson correlation analysis was performed using GraphPad Prism 7.0. Statistical analysis of all experimental data was performed using two-way ANOVA (* *p* < 0.05, ** *p* < 0.01, *** *p* < 0.001). Data were expressed as mean ± SEM. Signiﬁcance was set at *p* < 0.05. The hierarchical clustering in heatmap was performed using CRAN pheatmap package in RStudio (version 0.99.887).
